# Is the BCG Vaccine an Answer to Future Pandemic Preparedness?

**DOI:** 10.3390/vaccines10020201

**Published:** 2022-01-27

**Authors:** Nadia H. Khan, Nadine G. Rouphael, Lindsey R. Baden, Daniel S. Graciaa

**Affiliations:** 1 College of Agriculture and Life Sciences, Cornell University, Ithaca, NY 14850, USA; nhk33@cornell.edu; 2 Division of Infectious Diseases, Department of Medicine, Emory University School of Medicine, Atlanta, GA 30322, USA; dsgraci@emory.edu; 3 Division of Infectious Diseases, Brigham and Women’s Hospital (BWH), Harvard Medical School, Boston, MA 02115, USA; lbaden@bwh.harvard.edu

**Keywords:** COVID-19, BCG, pandemic preparedness

## Abstract

While the development of severe acute respiratory syndrome coronavirus 2 (SARS-CoV-2) vaccines was rapid, time to development and implementation challenges remain that may impact the response to future pandemics. Trained immunity via bacille Calmette-Guerin (BCG) vaccination (an antigen agnostic strategy) offers a potential intervention against future novel pathogens via an existing, safe, and widely distributed vaccine to protect vulnerable populations and preserve health system capacity while targeted vaccines are developed and implemented.

## 1. Introduction

Since emerging as a global pandemic, the severe acute respiratory syndrome coronavirus 2 (SARS-CoV-2) has caused more than 325 million cases of coronavirus disease 2019 (COVID-19) and over 5.5 million deaths globally (as of January 2022) [1]. The development of a vaccine, from concept to licensing, typically takes 10–15 years. This drawn-out process and potential challenges in implementation result in ongoing transmission and unacceptably high mortality in the midst of a pandemic [2]. Prior to COVID-19, the fastest documented vaccine development initiative was the mumps vaccine in 1967, which occurred over a four-year period (influenza vaccines such as for pandemic H1N1 2009 have a different development path due to the established seasonal vaccine development process). However, due to the immense and unprecedented pressure placed on healthcare systems by the COVID-19 pandemic, the SARS-CoV-2 vaccine development process was initiated in early January 2020. The United States Food and Drug Administration (FDA) issued an emergency use authorization (EUA) for the BNT162b2 vaccine from Pfizer-BioNtech, the mRNA-1273 vaccine from Moderna (in December 2020), and the Janssen adenoviral vector vaccine Ad26.COV2.S (in February 2021) as well as granting full approval for the BNT162b2 vaccine in August 2021. The World Health Organization (WHO) has listed multiple vaccines for emergency use, in addition to Pfizer BNT162b2, Moderna mRNA-1273, and Janssen Ad26.COV2.S, such as the AstraZeneca/Oxford viral vector vaccine (granted approval in February 2021). In addition, other viral vectors as well as proteins and inactivated vaccines against SARS-CoV-2 have received approval by the WHO and across many countries. Unfortunately, while the timeframe for COVID-19 vaccine development was relatively short, it could not prevent the loss of many millions of lives worldwide due to challenges in manufacturing and delivery.

The landscape of the pandemic and containment via immunization was further complicated by the emergence of SARS-CoV-2 variants of concern (such as Alpha, Beta, Gamma, Delta, Omicron, etc.) with an associated decrease in the neutralization capacity of authorized vaccines for some variants of concern [3,4,5]. The duration of the immune response to existing SARS-CoV-2 vaccines remains to be defined, with early data suggesting it persists for at least six months from vaccination and nine months from natural infection [6,7], though waning immunity prompted many countries to recommend additional doses of COVID-19 vaccines for the general population.

Inducing an efficient, long-term innate immune response shows promise in mitigating the high mortality associated with novel viruses and the lengthy vaccine development, testing, and deployment process. One such mechanism is trained immunity such as that conferred via bacille Calmette-Guerin (BCG) vaccination. Utilizing BCG vaccination in an antigen agnostic strategy provides an adjunct measure for pandemic preparedness.

## 2. BCG, Trained Immunity, and Clinical Infection

Trained immunity involves an immune response resulting from long-term reprogramming of innate immune cells. It typically occurs following exposure to a primary stimulus, thereby altering a subsequent secondary response to a different stimulus. One hundred years since its introduction, the live attenuated BCG vaccine is the oldest vaccine still in use, and the non-specific immunologic effect of BCG vaccination is a well-described example of trained immunity [8]. This immune response involves epigenetic signaling and is primarily mediated by monocytes/macrophages and natural killer (NK) cells [9]. The adaptive immune response induced via BCG vaccination involves CD4^+^ and CD8^+^ T cell activation and heightened levels of interferon gamma (IFN-γ), which increase the anti-mycobacterial activity exhibited by macrophages. IFN-γ also contributes to the activation of B cells and the generation of antigen-specific antibodies by plasma cells [9]. BCG vaccination leads to upregulation of immune-priming long-coding ribonucleic acid (RNA) and increased messenger RNA (mRNA) expression, and increased cytokine production by BCG-trained macrophages results in high levels of tumor necrosis factor alpha (TNF-α) and interleukin 6 (IL-6) promoters [10,11,12]. The duration of these responses is unclear but may last one year or longer [13]. These accumulating data provide a mechanistic insight into the role of BCG vaccination in inducing non-target immunity.

In the context of improved understanding of trained immunity induced by BCG, observational and clinical trial data suggest BCG vaccination could have an impact on outcomes related to non-mycobacterial infections [9]. Several studies have provided evidence of decreased infant mortality related to BCG vaccination via non-target protection from BCG against infections other than *Mycobacterium tuberculosis* [14,15,16,17]. Observational data from Guinea-Bissau indicate that infants diagnosed with acute lower respiratory tract infection were less likely to be BCG vaccinated, and that BCG vaccination at birth may reduce neonatal sepsis, respiratory infection, and mortality [15]. A trial in Uganda similarly found fewer incident infections during the first six weeks of life among 280 neonates who received BCG on the day of birth compared to the 280 who were vaccinated at six weeks (hazard ratio 0.71, 95% confidence interval 0.53–0.95) but no changes in production of inflammatory cytokines [16] A recent trial randomized over 1200 infants in Australia to receive either BCG-Denmark or no BCG at birth and found no difference in the proportion with lower respiratory tract infection episodes at one year of life (risk difference −3.2, 95% confidence interval −9.0–2.6) [17]. This suggests that the protective non-target effect of BCG is small in high-income settings. However, multiple factors may influence these discordant findings by context including environmental exposure to mycobacteria, maternal BCG status, infant birth weight, and overall infectious disease burden [18]. Additional investigation may better define the potential impact of the BCG vaccine on overall mortality and non-mycobacterial infectious diseases while characterizing the mechanisms of its non-target immune effects.

Importantly, BCG vaccination has also been shown to enhance immune response to other vaccines. For example, those who received the influenza vaccine after BCG demonstrated higher titers of hemagglutination-inhibiting antibodies compared to those who received the influenza vaccine after placebo, and BCG vaccination also appeared to influence cytokine production [19]. Similar responses have been observed for other vaccines in infants receiving the BCG vaccine, though there appears to be a complex interplay with other childhood vaccines, particularly non-live vaccines [20]. The non-target benefit of BCG may be most pronounced when the BCG vaccine is administered after non-live vaccines, as suggested in a secondary analysis from India that found that children who received BCG and diphtheria–tetanus–pertussis (DTP) vaccines at the same time or BCG after DTP experienced lower mortality at one year of age [21]. A global ecologic study suggests that pertussis incidence is substantially lower in countries with immunization programs that administer both BCG and diphtheria–tetanus–acellular pertussis (DTaP) vaccines compared to those that do not give BCG [22]. Indeed, some authors have suggested that discontinuing live vaccine administration after disease eradication could have unintended effects due to the loss of non-specific protection [23]. As new candidate tuberculosis (TB) vaccines are being developed, the WHO indicated that characterization of vaccine efficacy against all-cause pediatric mortality in addition to TB mortality should be considered [24]. While the timing of BCG vaccination may be important for overall trained immunity effects, as an intervention against a pandemic pathogen, it is likely to be the most recent vaccine administered, making timing less of a consideration. The phenomenon of trained immunity also extends to viral and parasitic infections, with evidence from studies of yellow fever and malaria [25,26]. In a randomized, placebo-controlled human trial, BCG vaccination was associated with decreased levels of viremia and upregulation of interleukin 1 beta (IL-1β), a heterologous cytokine responsible for trained immunity. It was found that BCG vaccine-induced epigenetic reprogramming of human monocytes conferred antigen agnostic protection against yellow fever [25]. BCG-induced trained immunity in healthy volunteers who underwent controlled malaria infection was associated with earlier expression of CD69 (a marker of activation) on NK cells, which in turn was correlated with lower *Plasmodium falciparum* parasitemia [26]. While an immune correlate of protection has yet to be defined, this developing evidence base for BCG-related trained immunity has prompted additional inquiry into non-target effects in clinical scenarios that may be relevant for future pandemics.

The recent ACTIVATE randomized trial of BCG vaccination in adults 65 years of age and older provides evidence that BCG is protective against a composite outcome of multiple common infections, primarily driven by a reduction in likely viral respiratory tract infections [27]. Hospitalized patients in Greece aged 65 or older received either BCG or placebo at hospital discharge, with planned follow-up for 12 months. Motivated by the COVID-19 pandemic, investigators conducted an interim analysis of 150 patients, which showed BCG vaccination increased the time to first subsequent infection (median 16 vs. 11 weeks) with a hazard ratio of 0.55 (95% confidence interval 0.31–0.97). In addition, they found increased production of TNF-α, IL-1β, and interleukin 10 (IL-10) by peripheral blood mononuclear cells in the BCG-vaccinated group. The finding of apparent protection against respiratory infection is comparable to a previous study of older adults in Indonesia who received monthly BCG vaccination or placebo for three months, with the vaccinated group having a lower frequency of acute upper respiratory tract infection over six months of follow-up [28]. However, these studies had different endpoints (lower vs. upper respiratory infection) that were not clearly defined. Consistent with the demonstrated safety of BCG vaccination in immunization campaigns, adverse events in the ACTIVATE trial did not differ between the groups. Separately, a systematic review found an increase in mild local and systemic reactions such as ulcer or lymphadenopathy but no reported serious adverse events in immunocompetent individuals who underwent re-vaccination with BCG [29]. A retrospective analysis of adults in the Netherlands did not find evidence of safety concerns among those with recent BCG vaccination prior to the COVID-19 pandemic or an association between BCG-related cytokine production and reported symptoms, though this study primarily evaluated respiratory infection symptoms in a short follow-up period [30].

With this background, after the emergence of SARS-CoV-2, multiple studies have evaluated the association between BCG vaccination and COVID-19. Several observational studies from early in the pandemic suggested that BCG vaccination coverage was associated with decreased COVID-19 infections and mortality both in individual countries and when pooling data across multiple countries [31,32,33,34]. Investigators in Japan found that BCG coverage of infants born in 1999–2002, 2004, and 2014 was higher in prefectures without reported COVID-19 infections than in those with a high prevalence of infection through May 2020 [31]. Notably, identifying prefectures with no reported COVID-19 infections is not representative of overall SARS-CoV-2 transmission during the pandemic. A global study suggested an inverse correlation between BCG coverage from 1980–2018 and COVID-19 mortality through late April 2020 but likely suffers from under-reporting in many countries [32]. Similar issues impact the generalizability of a comparable study analyzing World Health Organization data through late May 2020 [33]. Overall, these findings are limited by the ecologic nature of these studies and limited testing for and reporting of COVID-19 cases in many settings early in 2020; they have not been consistently reproduced with more complete data from later in the pandemic [35]. A study in Sweden found no difference in COVID-19 cases and hospitalizations between adult cohorts born before and after the discontinuation of routine newborn BCG vaccination [36]. Another study from the University of California San Diego did not find a significant negative correlation (*p* = 0.16) between the percentage of BCG coverage and COVID-19 mortality worldwide [35]. A recent case-control study in Canada showed no difference in odds of childhood BCG vaccination between COVID-19 cases and controls identified from samples at the same hospital microbiology laboratory (adjusted odds ratio 1.01, 95% confidence interval 0.84–1.21) [37]. Additionally, an Israeli study showed no statistically significant difference (*p* = 0.15) in the number of COVID-19 cases in the BCG-vaccinated group (361 [11.7%]) versus the unvaccinated group (299 [10.4%]) [38]. However, there were limitations in the methodology. Proof of BCG vaccination was not included in the selection of the control and experimental groups. The study also included people with unknown vaccination status and displayed significant selection bias by only including participants based on reported symptoms. The heterogeneity of these results highlights the complexity of studying non-target effects of BCG vaccination.

Despite the above caveats, recent data suggest the concept of BCG-induced immunity merits further prospective investigation [39]. While it seems unlikely that childhood receipt of BCG vaccine influences COVID-19 cases or outcomes in adults, given the impact of BCG on trained immunity, the effect of more recent vaccination in adults is worth considering and is the subject of multiple ongoing clinical trials in diverse settings (Table 1). These studies are evaluating the use of BCG for protection against COVID-19 among at-risk adults such as the elderly and healthcare workers and are being conducted in both TB-endemic and non-endemic locations in Africa, Australia, Europe, and North America. Early data from such trials are mixed and suggest contrasting results in BCG-naïve versus previously vaccinated populations [40]. Final published results will be critical in resolving this question and informing the potential use of BCG vaccine as an intervention against emerging pathogens.

## 3. BCG Implementation

A potential clinical benefit of the BCG vaccine for future pandemics will not be realized without programmatic implementation. Fortunately, widespread BCG vaccine distribution is feasible and economically viable; 100 million doses of BCG are already being manufactured annually, with scope for additional manufacturing capability [41]. Furthermore, the average cost of the BCG vaccine is $2–3 United States (US) per dose [42]. A model constructed with epidemiologic data from Indonesia estimated the incremental cost per quality adjusted life years gained from universal vaccination (ICER) as US $104. This was well below a cost-effectiveness threshold of US $175 proposed as triple the gross domestic product (GDP) per capita. Additional factors such as disease protection, vaccination costs, and case detection rates were considered [41]. As BCG vaccination could potentially mitigate adverse outcomes associated with SARS-CoV-2 variants and future pandemics, the ICER could decrease even further. Furthermore, the study notes that this ICER value could be applicable to other TB-endemic low- and middle- income countries as well. From a logistical standpoint, the BCG vaccine is well-established, with implementation in more than 157 countries worldwide [43]. Coverage exceeds 90% in 101 countries and less than 60% in only nine [42]. Additionally, it is not necessary for BCG to be stored frozen in the cold chain [44]. Thus, economic modeling and analysis of logistical factors suggest that BCG implementation is both cost-effective and amenable to scaling up in situations where pandemic-specific vaccines are not yet available for distribution. However, it will be essential to ensure that the supply of BCG and other vaccines for children is not interrupted so as not to recapitulate the lost progress against TB and other infectious diseases such as malaria and human immunodeficiency virus that has been documented during the COVID-19 pandemic [45]. The speed and variety of COVID-19 vaccine development has been remarkable. While the mRNA platform allowed for the development of vaccine within one year and enables the vaccine to be manufactured within weeks, these products require freezer storage for the cold chain [46]. Other platforms such as viral vector, inactivated, or protein subunit vaccines have also been essential in the quest to vaccinate the worldwide community. However, the global implementation of COVID-19 vaccines has been uneven to date due to both supply and distribution issues such as cold chain interruption [47]. This makes the BCG vaccine an appealing candidate as a tool for pandemic preparedness.

## 4. BCG for Future Pandemic Preparedness

**Figure 1:**
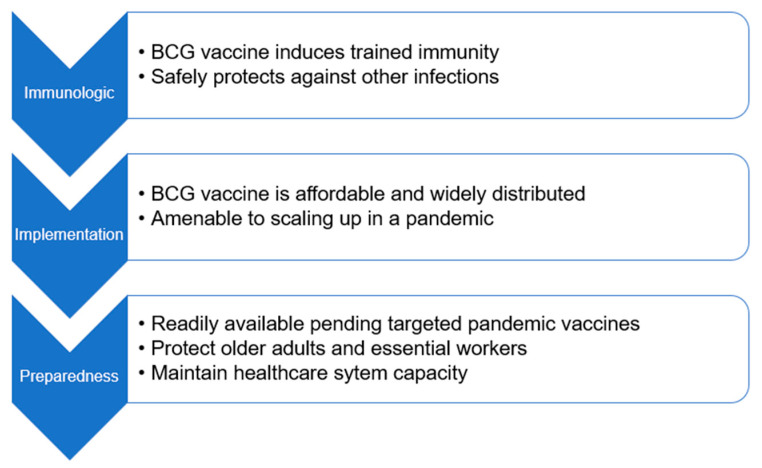
Immunologic & Implementation Rationale for BCG Vaccination for Pandemic Preparedness.

It is important to consider whether readily available products such as BCG or other live-attenuated vaccines could be utilized in the response to the next pandemic before a targeted vaccine is developed and implemented (Figure 1). BCG has immunologic, implementation, and preparedness advantages that position it well for such a strategy. Trained immunity induced by these vaccines could be particularly useful in protecting those most at risk during a public health crisis, including older adults with comorbid conditions, front-line workers, and healthcare personnel, both at an individual level and by reducing the healthcare system strain that has been evident throughout the COVID-19 pandemic. The BCG vaccine is safe, widely available, and relatively inexpensive and should be considered if targeted vaccines are not available. Indeed, modeling suggests this could have reduced COVID-19 mortality at a country level in early 2020 [48]. Data from ongoing prospective, randomized clinical trials of BCG and COVID-19 and respiratory illnesses in general may provide needed higher quality evidence for this strategy. In addition, investigators and funders should have trial platforms addressing this question ready to deploy for future pandemics so that it can be properly evaluated. BCG or other live vaccination could offer an important adjunct to non-pharmaceutical interventions against the next emerging infectious threat, particularly in the early stages of a pandemic. It would support the essential goal of protecting the most vulnerable and those who put themselves at risk to care for others either by decreasing mortality in the most vulnerable (such as the elderly and medically complex) or decreasing disease and staffing interruptions in the most needed workforce (such as healthcare workers).

Based on the data discussed above, we envision BCG as a supplemental strategy in addition to mRNA vaccines for SARS-CoV-2 in immunocompetent adults. The enhanced protection afforded by the BCG vaccine against novel variants or in times of increased community transmission may also have the advantage of preserving the workforce, specifically frontline healthcare professionals and other essential workers. A major limitation of the studies evaluating trained immunity and BCG is that the data are largely retrospective. Another limitation with the use of BCG vaccine as an adjunct strategy is the variability in the strains of BCG used in previous investigations, such as those evaluating childhood mortality. Evidence from a study of vaccinated infants suggests the BCG-Denmark strain (Statens Serum Institute) is more immunogenic than the BCG-Bulgaria or BCG-Russia strains, as measured by both BCG-stimulated and heterologous vaccine-stimulated responses, and that non-target effects may be strain-specific [49]. As a result, prospective trials are warranted to assess whether BCG-induced induction of trained immunity will diminish the morbidity and mortality of illness, thus obviating the need for more expensive strategies such as monoclonal antibody treatments that must be adapted to emerging variants, intensive care unit-level care, and mechanical ventilation while preserving the healthcare system capacity that continues to strain under the weight of a third year of a pandemic.

## 5. Conclusions

BCG vaccination induces broad immune effects that may provide protection against other pathogens. While the impact of BCG on COVID-19 remains to be fully characterized, the trained immunity resulting from BCG and other live vaccinations may provide an adjunctive approach to preventing illness from a range of pathogens, both existing and emerging. As COVID-19 continues to demonstrate, the world was not prepared to avoid devastating losses due to a new pathogen, and it is essential that we evaluate all potential tools to be better prepared for the next health threat.

## Figures and Tables

**Table 1:** Clinical Trials Evaluating BCG Vaccination and COVID-19 Listed on Clinicaltrials.gov (accessed on 27 November 2021).

Title	Locations	Identifier	N
BCG to Reduce Absenteeism Among Health Care Workers During the COVID-19 Pandemic	Cape Verde Guinea-Bissau Mozambique	NCT04641858	1050
Outcome of COVID-19 Cases Based on Tuberculin Test: Can Previous BCG Alter the Prognosis?	Egypt	NCT04347876	100
Use of BCG Vaccine as a Preventive Measure for COVID-19 in Health Care Workers	Brazil	NCT04659941	1000
Clinical Trial Evaluating the Effect of BCG Vaccination on the Incidence and Severity of SARS-CoV-2 Infections Among Healthcare Professionals During the COVID-19 Pandemic in Poland	Poland	NCT04659941	1000
Prevention, Efficacy and Safety of BCG Vaccine in COVID-19 Among Healthcare Workers	Mexico	NCT04461379	908
BCG Vaccine in Reducing Morbidity and Mortality in Elderly Individuals in COVID-19 Hotspots	India	NCT04475302	2175
Prevention of Respiratory Tract Infection and COVID-19 through BCG Vaccination in Vulnerable Older Adults	Netherlands	NCT04537663	5200
Application of BCG Vaccine for Immune-prophylaxis Among Egyptian Healthcare Workers During the Pandemic of COVID-19	Egypt	NCT04350931	900
Reducing COVID-19 Related Hospital Admission in Elderly by BCG Vaccination	Netherlands	NCT04417335	2014
BCG Against COVID-19 for Prevention and Amelioration of Severity Trial (BAC to the PAST)	United States	NCT04534803	2100
COVID-19: BCG As Therapeutic Vaccine, Transmission Limitation, and Immunoglobulin Enhancement	Brazil	NCT04369794	1000
BCG Vaccination for Healthcare Workers in COVID-19 Pandemic	South Africa	NCT04379336	500
Reducing Health Care Workers Absenteeism in COVID-19 Pandemic Through BCG Vaccine	Netherlands	NCT04328441	1500
BCG Vaccination to Protect Healthcare Workers Against COVID-19	Australia	NCT04327206	10078
Using BCG Vaccine to Protect Health Care Workers in the COVID-19 Pandemic	Denmark	NCT04373291	1293
Using BCG to Protect Senior Citizens During the COVID-19 Pandemic	Denmark	NCT04542330	1900
Efficacy of BCG Vaccination in the Prevention of COVID-19 via the Strengthening of Innate Immunity in Health Care Workers	France	NCT04384549	1120
BCG Vaccine for Health Care Workers as Defense Against COVID-19	United States	NCT04348370	1800

## Data Availability

Not applicable.
